# Predictors of patient-perceived functional improvement after cardiac resynchronization therapy: a real-world multi-dimensional analysis of clinical, imaging, and ECG predictors

**DOI:** 10.3389/fcvm.2026.1742350

**Published:** 2026-03-19

**Authors:** Ahmad Samir, Amr Yosry Emam, Rehab Elnagar, Shaimaa Nabil, Mahmoud Abdelfattah, Nevine Abdelmegeed, Eslam Ahmed, Omnia Kamel, Karimeldeen Hafez

**Affiliations:** 1 Cardiac Center Hail, Hail, Saudi Arabia; 2 Cardiology Department, Cairo University Kasr Alainy Faculty of Medicine, Cairo, Egypt; 3 Magdi Yacoub Heart Foundation, Cairo, Egypt; 4 Tanta University Faculty of Medicine, Tanta, Egypt; 5 Cardiology Department, Kafrelsheikh University, Kafr El-Shaikh, Egypt

**Keywords:** cardiac magnetic resonance CMR, Cardiac resynchronization therapy CRT, Functional improvement, Global longitudinal strain GLS, Heart failure with reduced ejection fraction HFrEF, NYHA functional class

## Abstract

**Introduction:**

Up to one-third of appropriately selected patients undergoing cardiac resynchronization therapy (CRT) fail to achieve meaningful clinical benefit despite fulfilling guidelines’ criteria. Traditional CRT response definitions [left ventricular (LV) end-systolic volume (ESV), ejection fraction (EF), and QRS duration] frequently diverge and occasionally fail to reflect patient-perceived clinical improvement.

**Objective:**

To identify predictors for patient-perceived functional improvement after CRT, incorporating various clinical, biochemical, electrocardiographic (ECG), and imaging variables. Functional improvement was defined as the changes in New York Heart Association (NYHA) functional class 6-9 months after CRT implantation.

**Methods:**

This retrospective single-center study included 97 heart failure (HF) patients meeting guideline-based CRT indications. Patients were categorized according to ≥1 NYHA class improvement at 6–9 months. Baseline and follow-up clinical, biochemical, ECG, echocardiographic, and cardiac magnetic resonance (CMR) parameters were compared between groups. Predictors of functional improvement were assessed using univariate and multivariate logistic regression.

**Results:**

Sixty-eight patients (70%) achieved ≥1 NYHA class improvement. At baseline, the non-improved group was characterized by higher NT-proBNP and creatinine levels, longer PR and shorter QTc intervals, and worse LV and RV global longitudinal strain (GLS) on CMR. At follow-up, NT-proBNP remained elevated in the non-improved group. Traditional CRT response criteria were more frequently met in the improved compared to the non-improved group (>15% LV ESV reduction: 94.7% vs. 5.3%, >10% LV EF improvement: 98.1% vs. 1.9%, and >20 ms QRS shortening: 86.2% vs. 13.8%); but none independently predicted functional improvement in multivariate analysis. Independent predictors for ≥1 NYHA class improvement included baseline renal function (OR 1.19, 95% CI 1.00–1.39, *p* = 0.034), baseline LV GLS (OR 0.62, 95% CI 0.41–0.96, *p* = 0.030), and follow-up E/A ratio (OR 0.28, 95% CI 0.09–0.87, *p* = 0.028).

**Conclusion:**

Functional improvement after CRT is best predicted by multi-dimensional assessment incorporating clinical, biochemical, and imaging parameters. In addition to the traditional response definitions, renal function, LV GLS, and LV diastolic indices were independently associated with a better patient-perceived functional improvement after CRT. Incorporating these factors into CRT eligibility and prognostication may enhance patient selection and improve outcomes.

## Graphical Abstract

**Figure:**
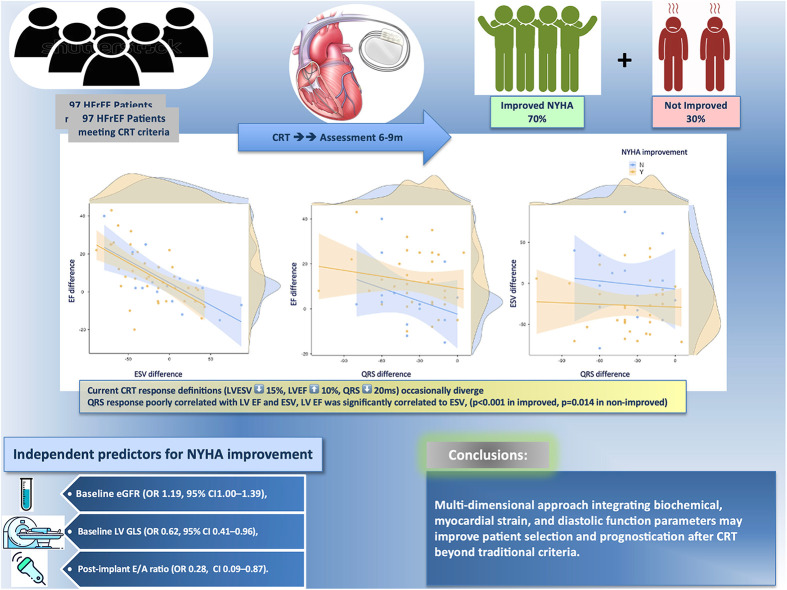

## Introduction

Heart failure (HF) is a complex clinical syndrome that is associated with substantial morbidity and mortality (1). Progressive decline in left ventricular (LV) function triggers compensatory mechanisms that lead to several adverse remodeling and structural changes within the LV, with subsequent electrical dyssynchrony manifesting on the 12-lead electrocardiogram (ECG) (2).

In the spectrum of heart failure with reduced ejection fraction (HFrEF), cardiac resynchronization therapy (CRT) has become a cornerstone treatment for eligible HFrEF patients who demonstrate electrical dyssynchrony (3, 4). Current guidelines consider CRT for HFrEF patients with ejection fraction (EF) ≤35%, QRS > 130 milliseconds (ms), and persistent symptoms despite optimal medical therapy (3–5). Major clinical trials such as MIRACLE (6), COMPANION (7), CARE-HF (8), and MADIT-CRT (9) have demonstrated that CRT improves symptoms, quality of life, and reduces hospitalization and mortality in appropriately selected HFrEF patients. However, despite fulfilling guideline criteria, a significant proportion of patients (reaching approximately 40%) fail to derive a meaningful clinical benefit after CRT (10). This alarming non-responding rate underscores that further refinement of the current candidacy criteria is critically needed.

Multiple definitions have been proposed to indicate “appropriate response” after CRT implantation, including favorable changes in the LV end-systolic volume (ESV), LV ejection fraction (EF), QRS duration, or other variables (11–13). This heterogeneity in response definitions and thresholds reflects the substantial variation in LV recovery profiles among different patients. It is now well-established that different patients may show non-uniform or occasionally discordant changes in the various parameters approved to indicate CRT-response. Nevertheless, functional improvement –expressed in NYHA class change—is arguably the most meaningful from the patient perspectives, directly reflecting symptomatic benefit as compared to other sophisticated numerical values.

Accordingly, this study systematically evaluated baseline and follow-up clinical, biochemical, electrocardiographic (ECG), echocardiographic, and cardiac magnetic resonance (CMR) parameters in a cohort of HFrEF patients who received CRT in accordance with Class I guidelines’ recommendations (3, 4). The study aimed to elucidate the key correlates and predictors of clinically meaningful improvement in NYHA functional class following CRT implantation.

## Methods

This retrospective, single-center, observational-analytic study reviewed the institutional database for all patients who had received CRT as part of the guidelines-directed medical therapy (GDMT) between January 2015 and June 2024. Patients were included in the analysis if they were: (1) ≥18 years old; (2) receiving GDMT for ≥42 days; and (3) meeting guidelines-based indications for CRT; [LVEF ≤ 35%, left bundle branch block (LBBB) morphology with QRS duration ≥ 130 ms, and NYHA Class II–ambulant IV despite optimal GDMT]. Exclusion criteria were: (1) having a clear, non-cardiac morbidity indicating an estimated life expectancy <2 years, or (2) Non-LBBB morphology in pre-implantation assessment. As per institutional protocols, CRT-eligible patients underwent CMR within 2 months prior to the scheduled implantation.

According to the post-implantation clinical assessments, patients were stratified into two groups based on the NYHA class category consistently achieved by 6-9 months post-implantation. The “improved NYHA” indicated those who showed improvement by ≥1 class compared to pre-implantation, while the “non-improved” indicated those reporting unchanged or worsened NYHA class. Baseline demographic, clinical, biochemical, ECG, echocardiographic, and CMR data were extracted from the electronic medical records of the visits prior to CRT implantation. Similarly, follow-up data were obtained from the assessments conducted 6-to-9 months post-implantation, according to the institutional protocols.

### Response definitions

Functional improvement was defined as a consistent self-reported improvement of ≥1 NYHA class compared to pre-implantation status. Patients were asked to describe their functional capacity in reproducible, activity-based terms. NYHA class 4 indicated reporting shortness of breath (SOB) at rest, class 3 indicated reporting SOB or significant limitations while moving between in-house rooms, class 2 indicated SOB on climbing one flight of stairs, and class 1 indicated the ability to climb seamlessly more than one flight of stairs. To ensure reproducibility and minimize assessment variability, these standardized questions were consistently applied across all patient encounters. NYHA assessments were conducted by clinicians following institutional protocols, with patients asked to describe their current functional capacity using these specific activity-based criteria. The retrospective nature of data collection precluded formal blinding to other clinical parameters; however, NYHA classifications were documented during routine clinical visits prior to any study-related analysis, thereby preventing intentional bias in outcome adjudication.

### Protocolized management for medical and device therapy

All patients received guideline-directed medical therapy (GDMT) according to institutional heart failure protocols. Medical management included systematic uptitration of neurohormonal antagonists (angiotensin-converting enzyme inhibitors/angiotensin receptor blockers or angiotensin receptor-neprilysin inhibitors, beta-blockers, and mineralocorticoid receptor antagonists) to target or maximally tolerated doses, along with diuretics as needed for volume management. GDMT optimization was standardized across all patients through regular heart failure clinic follow-up visits concurrent with device interrogation and optimization. Additionally, periodic post-implantation visits were aligned for periodic device interrogation and optimization of pacing parameters.

According to the most rigorously validated evidence from published landmark trials, the threshold for LV ESV adequate response was defined as a relative reduction of the ESV by ≥15% compared to the pre-implantation assessment, for LV EF was defined as an increase by absolute ≥10%, and for QRS duration was defined as shortening of the QRS by ≥20 ms in a 12-lead ECG (14–17). The LV function and volumes were assessed by averaging ≥3 loops taken and evaluated by bi-plane Simpson's method with careful exclusion of any loops containing premature beats or significant respiratory movements. In addition to the validated binary definitions, the magnitudes of changes in the ECG and echocardiographic, as well as biochemical, and CMR parameters were contrasted as continuous variables. CMR examinations were performed using a 1.5 T MRI scanner with standard steady-state free precession (SSFP) cine sequences in long-axis views and short-axis stacks. Volumetric analysis and strain quantification were performed using Segment software version 4.0.1 (Medviso AB, Lund, Sweden) (18). Endocardial and epicardial borders were manually contoured on end-diastolic frames with automated tracking throughout the cardiac cycle. LV GLS was calculated by averaging longitudinal strain from 2-, 3-, and 4-chamber views. LV GCS was derived from short-axis images. RV strain parameters were similarly obtained from 4-chamber views (GLS) and short-axis images (GCS). All analyses were performed by experienced observers blinded to clinical outcomes, with inter-observer ICC >0.85 for LV GLS measurements.

### Ethical approval and patient confidentiality

The study protocol, methodology, and results were reviewed and approved by the institutional ethics committee (registration number 20241031MYFAHC_CRTFP20250213). All collected data were anonymized and were subjected to statistical analysis after full concealing of patients’ identifiers. Statistical analysis and results were performed on the anonymized collected data. Given the retrospective nature of the analysis and the complete preservation of patients’ confidentiality, the institutional ethical committee approved waiving of patient consent for participation and publication.

### Sample size and statistical analysis

This study employed a convenience sampling approach, analyzing all consecutively enrolled patients who met eligibility criteria during the study period. While a formal *a priori* sample size calculation was not performed, our cohort of 97 patients with 68 events (NYHA improvement) provides adequate statistical power for the primary analyses.

Statistical analyses were conducted using SPSS version 27.0 (IBM Corp., Armonk, NY) and Jamovi statistics version 2.3 (The Jamovi project, 2022). Categorical variables were represented as frequencies (percentages) and compared between groups using Chi-square/Fisher's exact tests. Continuous variables were first subjected to normality testing by Shapiro–Wilk and histograms, then according to the distribution profiles, were expressed as means (standard deviation) or medians (25th—75th percentiles, indicating the interquartile range IQR), as appropriate. Subsequently, between-group comparisons for continuous variables used Student's t-test or Mann–Whitney U test as appropriate. Univariate binary logistic regression was designed to recognize potential predictors for NYHA improvement after CRT implantation. Variables demonstrating statistical significance (*p* ≤ 0.05) or a strong trends toward significance (*p* < 0.10) in univariate logistic regression were considered as candidates for multivariate analysis. Multivariate regression models (by ENTER method) were designed to determine independent predictors. To adhere to the guidelines of maintaining a minimum events-per-variable (EPV) ratio, separate models were constructed for baseline and follow-up parameters to avoid model overfitting given the limited number of non-responders (*n* = 29). The odds ratio (OR) and 95% confidence interval (CI) were identified for significant predictors. Overall model performance was evaluated via receiver operating characteristic (ROC) curves. Data completeness was high across most variables. Missing data were minimal (<5% for most variables) and primarily limited to CMR-derived strain parameters. Complete-case analysis was employed for all statistical comparisons, as missing data were sporadic and considered missing completely at random (MCAR). A p-value of ≤0.05 was considered the threshold for a statistically significant difference between groups.

## Results

A total of 97 patients met the eligibility criteria and were included in the analysis. The mean age was 52.4 ± 13.9 years, 65 (67%) were male, and ischaemic cardiomyopathy was the pathology in 38 patients (39%). At 6–9 months follow-up, 70% of patients (*n* = 68) demonstrated a consistent ≥1 NYHA class improvement (‘improved’ group), while 30% (*n* = 29) showed no change or worsening NYHA class (‘non-improved’ group). The median follow-up period was 36 months (IQR 15-72 months).

Careful assessment of the baseline features showed that age, sex, body mass index (BMI), baseline HF status, and comorbidity profiles were comparable between the 2 groups. However, the non-improved group was characterized by significantly higher NT-proBNP (3146 vs. 1341 pg/mL, *p* = 0.011), higher serum creatinine (1.0 vs. 0.7 mg/dL, *p* = 0.001), lower eGFR (102.9 vs 78.8 mL/min/m^2^, *p* = 0.004), longer PR interval (190 vs. 180 ms, *p* = 0.005), shorter QTc interval (459 vs. 491 ms, *p* = 0.002), and worse CMR-feature tracking-derived LV GLS (−4.6% vs. −6.25%, *p* < 0.001) and RV GLS (−4.4% vs. −6.7%, *p* = 0.035), compared to the improved group. The baseline pre-implantation characteristics are demonstrated in Table 1.

**Table 1:** Baseline characteristics of the two subgroups.

Variable	Improved NYHA (*n* 68)	Non-improved NYHA(*n* 29)	*p*-value
Demographic and clinical variables:			
Age, years	52.0 [42, 60.0]	49.5 [38, 60.0]	0.32
Female sex	26 (38.2%)	6 (20.7%)	0.105
Weight, kg	84.0 ± 18.9	80.6 ± 21.6	0.387
Height, cm	167 ± 7.8	168.5 ± 9.4	0.229
BMI, kg/m^2^	30.4 ± 5.9	28.3 ± 6.6	0.114
Smoking status:			0.488
Non-smoker	46 (67.7%)	10 (34.4%)	
Current smoker	12 (17.6%)	5 (17.2%)	
Hypertension	34 (64.2%)	19 (35.8%)	0.148
Diabetes mellitus	42 (77.8%)	12 (22.2%)	0.16
NT-proBNP, pg/mL	1341 [520, 2273]	3146 [1649, 5291]	**0**.**011**
Serum Creatinine, mg/dL	0.7 [0.6, 1.05]	1.0 [0.8, 1.3]	**0**.**001**
eGFR, mL/min/1.73m^2^	102.9 [78.9, 125.0]	78.8 [65.2, 104.0]	**0**.**004**
6MWT, meters	260 [198, 330]	273 [198, 330]	0.645
Baseline NYHA			0.23
Class 2	15 (29%)	11 (44%)	
Class 3	35 (67%)	14 (52%)	
Class 4	1 (2%)	0 (0%)	
Electrocardiographic:			
PR, ms	180 ± 25.2	190 ± 15.9	**0**.**005**
QRS duration, ms	160 ± 17.0	163 ± 19.6	0.219
QTc, ms	491 ± 28.7	459 ± 27.2	**0**.**002**
Echocardiography:			
LV EDD, cm	6.8 ± 1.36	7.05 ± 1.18	0.987
LV ESD, cm	6.0 ± 1.29	6.35 ± 1.25	0.825
LV ESV, mL	180 [113, 255]	205 [160, 262]	0.455
LV EF, %	28 ± 9.3	24 ± 9.9	0.307
LA diameter, cm	4.5 ± 0.76	4.4 ± 0.72	0.422
E/A	1.4 [0.9, 2.5]	1.75 [0.75, 3.65]	0.97
E/E'	14.5 ± 3.7	15.0 ± 2.6	0.611
TAPSE, mm	20 ± 5.6	18 ± 5.4	0.703
RV S', cm	10.4 ± 2.2	10.9 ± 3.9	0.671
PASP, mmHg	32 [25, 47]	32 [28, 51]	0.748
Cardiac Magnetic Resonance imaging and feature tracking:			
LV EF, %	23 ± 11.1	20 ± 12.2	0.556
LV EDV, mL	319 [267, 421]	298 [253, 486]	0.834
LV ESV, mL	240 [170, 421]	223 [160, 423]	0.702
LV SV, mL	76 ± 26.6	72 ± 42.3	0.625
RV EF, %	45 ± 17.5	38.5 ± 16.4	0.189
RV EDV, mL	147 [123, 186]	155.5 [121, 264]	0.41
RV ESV, mL	91 [54.5, 117]	76.5 [54, 194]	0.842
RV SV, mL	65 ± 24.6	65 ± 36.6	0.95
Cardiac output, L/min	5.0 ± 1.3	5.1 ± 1.0	0.855
LV mass, g	113 ± 51.3	134.5 ± 62.9	0.158
Myocardial scar, %	0 [0, 6.5]	0 [0, 8.4]	0.312
LV GLS, %	−6.25 ± 3.1	−4.6 ± 1.9	**< 0**.**001**
LV GCS, %	−5.4 ± 3.3	−6.2 ± 3.8	0.316
RV GLS, %	−6.7 ± 4.9	−4.4 ± 3.6	**0**.**035**
RV GCS, %	−3.0 ± 3.2	−3.0 ± 2.8	0.856

Data expressed as frequency (percent), mean ± standard deviation, or median [25th, 75th percentile]. Bold *p-values* indicate statistically significant. 6MWT, 6-minute walk test; BMI, body mass index; CMR, cardiac magnetic resonance; EDD, end-diastolic diameter; EDV, end-diastolic volume; EF, ejection fraction; eGFR, estimated glomerular filtration rate; ESD, end-systolic diameter; ESV, end-systolic volume; GCS, global circumferential strain; GLS, global longitudinal strain; LA, left atrial; LV, left ventricular; ms, milliseconds; NT-proBNP, N-terminal pro B-type natriuretic peptide; NYHA, New York Heart Association; PASP, pulmonary artery systolic pressure; QRS, QRS complex duration; QTc, corrected QT interval; RV, right ventricular; SV, stroke volume; TAPSE, tricuspid annular plane systolic excursion.

At 6-9 months follow-up evaluation, the non-improved group had significantly higher NT-proBNP (2152.5 vs. 640 pg/mL, *p* = 0.006). Post-implantation changes in LV ESV, LV EF, and QRS showed numerically favorable trends in the improved group, although they did not reach statistical significance. Diastolic parameters (E/A and E/e’) showed statistical trends towards worse levels in the non-improved group. The post-implantation follow-up data are detailed in Table 2. All established binary CRT response criteria (namely LV ESV reduction ≥15%, LV EF increase ≥10%, and QRS shortening ≥20 ms) were more frequently achieved in the improved compared to the non-improved patients (all *p* < 0.001).

**Table 2:** Post-implantation follow-up characteristics for the study subgroups.

Variable	Improved NYHA	Non-improved NYHA	*p*-value
Biomarkers:			
NT-proBNP (pg/mL)	640 [199, 1620]	2152.5 [961, 3392]	**0**.**006**
Change in NT-proBNP (%)	−28 [−77, +27]	−16 [−57, +51]	0.81
Electrocardiography:			
PR, ms	130 ± 27.9	130 ± 29.3	0.491
QRS duration, ms	120 ± 20.9	130 ± 18.8	0.184
QRS difference, Δ ms	−37.9 ± 23.8	−35 ± 28.6	0.36
QTc, ms	468 ± 42.8	472.5 ± 44.3	0.785
Percent of biventricular pacing, %	98 ± 2.9	99 ± 2.0	0.795
Echocardiography:			
LV EF, %	37 [26, 44]	30 [21, 35]	0.091
LV EDD, cm	6.7 ± 1.6	7.6 ± 1.8	0.448
LV ESD, cm	5.5 ± 1.8	6.4 ± 2.0	0.456
LV ESV, mL	147 [78.6, 216]	208.5 [119, 321]	0.286
E/A	1.1 ± 0.91	2.8 ± 1.6	0.055
E/e’	12.0 ± 5.6	17.5 ± 6.3	0.067
RV ś	12.0 ± 2.9	10.9 ± 2.4	0.644
TAPSE, mm	20.5 ± 4.9	21.0 ± 4.7	0.574
PASP, mmHg	33.0 ± 16.2	32.0 ± 9.7	0.869
EF difference, Δ	9.4 ± 14.1	4.9 ± 14.3	0.061
ESV difference, Δ mL	−23.6 ± 35	−0.8 ± 43.9	**0**.**046**
Response Rates^a^:			
QRS response	50 (86.2%)	8 (13.8%)	**0**.**002**
LV EF response	53 (98.1%)	1 (1.9%)	**<0**.**001**
LV ESV response	54 (94.7%)	3 (5.3%)	**<0**.**001**

Data expressed as frequency (percent), mean ± standard deviation, or median [25th, 75th percentile]. Bold *p-values* indicate statistically significant. 6MWT, 6-minute walk test; BMI, body mass index; CMR, cardiac magnetic resonance; EDD, end-diastolic diameter; EDV, end-diastolic volume; EF, ejection fraction; eGFR, estimated glomerular filtration rate; ESD, end-systolic diameter; ESV, end-systolic volume; GCS, global circumferential strain; GLS, global longitudinal strain; LA, left atrial; LV, left ventricular; ms, milliseconds; NT-proBNP, N-terminal pro B-type natriuretic peptide; NYHA, New York Heart Association; PASP, pulmonary artery systolic pressure; QRS, QRS complex duration; QTc, corrected QT interval; RV, right ventricular; SV, stroke volume; TAPSE, tricuspid annular plane systolic excursion.

^a^ Binary response rates defined as reduction by ≥20 ms for QRS duration, absolute increase by ≥10 points in LV EF, and relative decrease by ≥15% in LV ESV, respectively.

### Correlations between different Crt-response definitions

**Figure 1:**
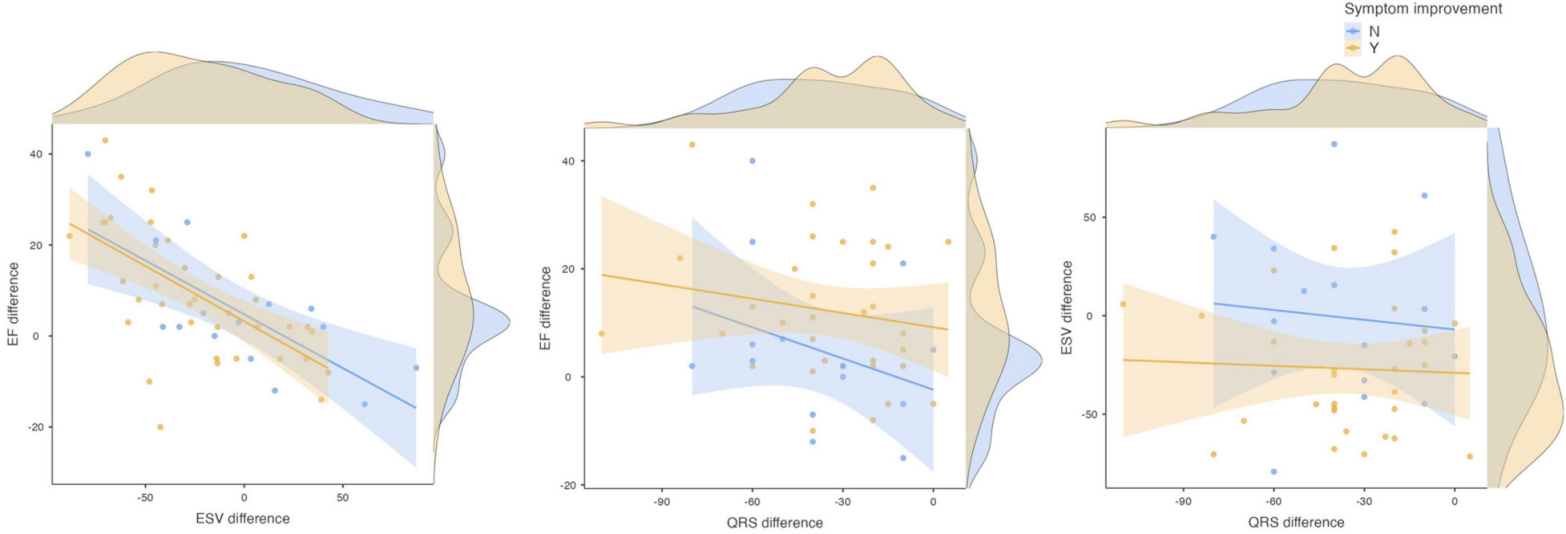
Correlation matrix scatterplot for the LV EF difference, LV ESV difference, and QRS duration difference after CRT-implantation, categorized by ≥1 NYHA class symptoms improvement. EF, ejection fraction; ESV, end-systolic volume; LV, left ventricle.

Correlations between the quantitative changes in CRT response parameters [i.e., pre- to post-implantation change (Δ) in LV ESV, in LV EF, and in QRS duration] were first assessed in the whole cohort, then were repeated with stratifying patients according to the NYHA improvement endpoint. Changes in QRS duration showed no significant correlation with either LV EF improvement (r = −0.176, *p* = 0.334) or LV ESV reduction (r = 0.034, *p* = 0.858), highlighting discordance between electrical and mechanical response parameters (Table 3 and Figure 1). This lack of correlation persisted when stratifying patients by NYHA improvement status. In contrast, LV EF and LV ESV changes demonstrated strong inverse correlation in all groups (whole cohort: r = −0.641, *p* < 0.001; improved group: r = −0.641, *p* < 0.001; non-improved group: r = −0.620, *p* = 0.014), confirming coherent mechanical remodeling patterns that can be independent of electrical changes.

**Table 3:** Correlations between CRT-response definitions.

Correlation pair	Group	Spearman's rho (r)	*p*-value
EF diff vs. QRS diff	All patients	−0.176	0.334
	Improved	−0.176	0.334
	Non-Improved	−0.357	0.191
EF diff vs. ESV diff	All patients	−0.641	**<0.001**
	Improved	−0.641	**<0.001**
	Non-Improved	−0.620	**0**.**014**
QRS diff vs. ESV diff	All patients	0.034	0.858
	Improved	0.034	0.858
	Non-improved	−0.167	0.552

BMI, body mass index; CMR, cardiac magnetic resonance; EDD, end-diastolic diameter; EDV, end-diastolic volume; EF, ejection fraction; eGFR, estimated glomerular filtration rate; ESD, END-systolic diameter; ESV, end-systolic volume; GCS, global circumferential strain; GLS, global longitudinal strain; LA, left atrial; LV, left ventricular; ms, milliseconds; NT-proBNP, N-terminal pro B-type natriuretic peptide; NYHA, New York Heart Association; PASP, pulmonary artery systolic pressure; QRS, QRS complex duration; QTc, corrected QT interval; RV, right ventricular; SV, stroke volume; TAPSE, tricuspid annular plane systolic excursion. Bold *p-values* indicate statistically significant. diff, difference between baseline and follow-up.

### Univariate and multivariate analysis to recognize significant predictors

Logistic univariate regression analysis was conducted to determine significant predictors for NYHA improvement. Significant predictors for NYHA improvement from baseline characteristics included renal functions, QTc, and CMR-feature tracking-derived LV GLS. Likewise, post-implantation predictors included renal functions, LV EDD, LV ESD, and E/A ratio. The univariate regression is represented in Table 4.

**Table 4:** Univariate regression analysis for NYHA improvement.

Predictor	Odds ratio	95% CI lower	95% CI upper	*p*-value
Baseline predictors				
Age at implantation	0.982	0.95	1.02	0.297
BMI	1.028	0.9444	1.12	0.528
Serum creatinine_baseline	0.184	0.0486	0.695	**0**.**013**
eGFR_baseline	1.024	1.0068	1.04	**0**.**006**
QRS d_baseline	1.01	0.98078	1.04	0.515
QTc _baseline	1.03	1	1.047	**0**.**015**
LV EDD_baseline	0.81	0.537	1.22	0.315
LV ESD_baseline	0.788	0.515	1.21	0.273
LV ESV_baseline	0.998	0.992	1	0.399
LV EF_baseline	1.02	0.963	1.09	0.458
TAPSE_baseline	1.059	0.9257	1.21	0.404
RV S’ _baseline	1.03	0.7166	1.48	0.87
LV EF_CMR_baseline	1.02	0.976	1.07	0.341
LV EDV_CMR_baseline	0.999	0.996	1	0.742
LV ESV_CMR_baseline	1	0.996	1	0.839
LV SV_CMR_baseline	0.998	0.983	1.01	0.817
RV EF_CMR_baseline	1.026	0.994	1.06	0.118
RV EDV_CMR_baseline	0.997	0.991	1	0.41
RV ESV_CMR_baseline	0.996	0.989	1	0.228
LV GLS_CMR_baseline	0.697	0.5344	0.909	**0**.**008**
RV GLS_CMR_baseline	0.99	0.876	1.12	0.865
Post-implantation predictors				
PR _by follow-up	0.991	0.973	1.01	0.373
QRS d _by follow-up	0.999	0.975	1.02	0.945
QTc _by follow-up	0.993	0.982	1	0.231
Serum creatinine_by follow-up	0.344	0.129	0.915	**0**.**032**
eGFR _by follow-up	1.014	1.001	1.03	**0**.**03**
LV EDD _by follow-up	0.694	0.485	0.992	**0**.**045**
LV ESD _by follow-up	0.729	0.532	1	**0**.**05**
LV ESV _by follow-up	0.996	0.992	1	0.108
LV EF _by follow-up	1.032	0.989	1.08	0.148
E/A _by follow-up	0.416	0.203	0.855	**0**.**017**
E/e’ _by follow-up	0.927	0.816	1.05	0.24
TAPSE _by follow-up	1.08	0.9234	1.26	0.336
RV S’ _by follow-up	1.097	0.7921	1.52	0.577
LV EF _by follow-up	1.032	0.989	1.08	0.148
Percent change NT-pro-BNP	1.06	0.784	1.44	0.695
LV EF difference	1.02	0.979	1.07	0.303
LV ESV difference	0.985	0.969	1	0.063
QRS difference	0.995	0.975	1.02	0.658

CI, confidence interval; BMI, body mass index; CMR, cardiac magnetic resonance; EDD, end-diastolic diameter; EDV, end-diastolic volume; EF, ejection fraction; eGFR, estimated glomerular filtration rate; ESD, END-systolic diameter; ESV, end-systolic volume; GCS, global circumferential strain; GLS, global longitudinal strain; LA, left atrial; LV, left ventricular; ms, milliseconds; NT-proBNP, N-terminal pro B-type natriuretic peptide; NYHA, New York Heart Association; PASP, pulmonary artery systolic pressure; QRS, QRS complex duration; QTc, corrected QT interval; RV, right ventricular; SV, stroke volume; TAPSE, tricuspid annular plane systolic excursion. Bold *p-values* indicate statistically significant.

Two multivariate regression models were constructed; one model focused on baseline characteristics and another for the post-implantation parameters. In the first model, baseline eGFR (OR: 1.19, 95% CI: 1.0–1.39, p: 0.034) and the CMR-derived LV GLS (OR: 0.62, 95% CI: 0.41–0.96, p: 0.03) were independent predictors for NYHA improvement. In the second model assessing the post-implantation parameters, E/A ratio was a significant independent predictor (OR: 0.28, 95% CI: 0.09–0.87, p: 0.028), while the follow-up eGFR had a borderline trend for significance (OR: 1.04, 95% CI: 0.99–1.08, p: 0.06).

## Discussion

This study provides important patient-centered insights from real-world practice into predictors of functional improvement following CRT in appropriately selected HFrEF patients. Three key findings emerge from the analysis of the 97 patients included in this study. First, despite fulfilling guidelines’ eligibility criteria, nearly one-third of patients do not derive meaningful functional improvement after CRT. Second, conventional CRT response metrics (LV ESV reduction, LV EF improvement, and QRS narrowing) are often discordant and correlate poorly with patient-perceived functional benefits. Third, a multi-dimensional approach incorporating biochemical, myocardial strain, and diastolic function indices may predict symptomatic improvement better than relying solely on the current conventional criteria.

### Biochemical predictors: the cardiorenal axis

The study findings demonstrate that both baseline and follow-up NT-proBNP levels, along with renal function (represented by serum creatinine and eGFR), are robust predictors of functional non-response. Patients with poor functional outcomes demonstrated significantly higher baseline NT-proBNP (3,146 vs. 1,341 pg/mL) and impaired renal function (eGFR 78.8 vs. 102.9 mL/min/1.73 m^2^). This aligns with previous studies showing the prognostic value of natriuretic peptides and renal function in CRT outcomes (8, 19–24).

Mechanistically, markedly elevated baseline NT-proBNP reflects advanced myocardial wall stress that might have exceeded the point of reversibility by electrical re-synchronization (25). On the other hand, the persistence of elevated NT-proBNP despite CRT implantation likely identifies patients in whom the intervention failed to reverse the fundamental pathophysiologic processes and adverse remodeling driving heart failure progression.

Similarly, renal dysfunction represents a manifestation of the complex cardiorenal syndrome, where impaired kidney function may limit CRT effectiveness through the unmanageable neurohormonal activation, the reduced tolerance to GDMT, as well as the persistent volume overload and myocardial stretch (26, 27). Also, concomitant chronic renal impairment frequently challenges adherence to GDMT in HFrEF patients with repeated transient discontinuations or dose reductions, particularly for renin angiotensin system-modulating therapies (28).

### Electrocardiographic insights: beyond Qrs duration

Prolonged baseline PR interval was associated with a reduced likelihood of functional improvement, consistent with prior evidence suggesting that first-degree AV block may reflect intrinsic dyssynchrony patterns less amenable to improvement with CRT (29, 30). Prior studies have shown that patients with prolonged PR intervals may represent a subgroup with a lesser intrinsic ventricular (Purkinje-mediated) desynchrony at baseline, thus limiting potential gains from resynchronization therapy (30). Interestingly, a shorter baseline QTc interval was more common in the non-improved subgroup, which may indicate underlying myocardial scarring or altered repolarization dynamics (31), though this observation requires further validation in larger cohorts.

### Advanced imaging: the promise of strain analysis

In this study, CMR-derived LV GLS by feature tracking analysis emerged as a robust independent predictor of functional improvement, supporting the growing evidence that myocardial deformation indices are more sensitive than the two-dimensional-derived LV EF in reflecting contractile reserve (32–34). Findings of the present study align with a recent systematic review and meta-analysis of 3,981 patients demonstrating that worse baseline LV GLS predicts poor post-CRT outcomes (33, 35). Compared to echocardiography-derived speckle tracking to evaluate myocardial strain, CMR-based strain analysis overcomes the common limitations, including variable acoustic windows and challenges in endocardial border tracing, in addition to its superiority in spatial resolution and reproducibility (33, 36). Additionally, the predictive value of RV GLS highlights the importance of biventricular assessment in CRT candidacy, as RV dysfunction was found to significantly influence adverse outcomes in HFrEF patients (32).

### Diastolic function: the underappreciated facet

In this study, post-implantation E/A ratio emerged as a significant independent predictor for NYHA improvement in multivariate analysis, underscoring a crucial yet often overlooked role for diastolic metrics in post-CRT outcomes. This finding carries important mechanistic and clinical implications. Restrictive filling patterns and significantly elevated filling pressures reflect advanced structural remodeling, myocardial fibrosis, and impaired ventricular compliance—pathophysiologic changes that, if irreversible, would substantially limit symptomatic benefits even when systolic indices demonstrate improvement (37, 38). The independent predictive value of E/A ratio suggests that the degree of diastolic impairment and its potential reversibility may be as important as systolic function in determining patient-perceived functional recovery.

While other diastolic parameters assessed in this study (E/e’ ratio, LA dimensions, and pulmonary artery pressures) did not independently predict NYHA improvement, this finding should be interpreted with appropriate caution rather than dismissing their potential relevance. Comprehensive diastolic function grading in advanced HFrEF populations is inherently complex and technically challenging, particularly when confounded by the constellation of findings prevalent in our cohort: severe LV dysfunction, significant functional mitral regurgitation, tricuspid regurgitation, and secondary pulmonary hypertension. These comorbid conditions can obscure accurate classification using conventional diastolic dysfunction grading schemes and may introduce measurement variability that reduces the discriminatory power of individual parameters. Furthermore, the relatively modest sample size and the predominance of advanced disease in both groups may have limited our ability to detect more subtle associations.

Despite these limitations, our findings suggest that diastolic assessment should be systematically integrated into CRT response evaluation and may potentially inform patient selection criteria. The exact role of baseline diastolic dysfunction severity, the patterns of diastolic parameter changes post-CRT, and the threshold beyond which diastolic impairment becomes irreversible all remain important questions requiring comprehensive evaluation. Future prospective studies with larger cohorts should incorporate multimodal diastolic assessment—including both non-invasive echocardiographic indices and, when clinically appropriate, invasive hemodynamic measurements of ventricular filling pressures—to fully elucidate the relationship between diastolic function and patient-centered outcomes after CRT.

### Rethinking the current response definitions and outcome prediction

Our analysis highlights the frequent divergence between electrocardiographic (QRS narrowing) and echocardiographic (LV EF and LV ESV changes) response metrics. This frequently observed divergence emphasizes the complex and multifaceted nature of LV recovery patterns and remodeling after CRT (39). In this cohort, although all conventional binary definitions for adequate CRT response (namely, ≥15% LV ESV reduction, ≥10% LV EF improvement, and ≥20 ms QRS shortening) were significantly more prevalent among patients who reported NYHA improvement, none independently predicted symptomatic recovery in multivariate analysis.

The observed discordance between conventional CRT-response definitions and patient-perceived benefit underscores the need for alternate endpoints that better align with clinically meaningful outcomes. In this context, several efforts have been made to develop predictive models that incorporate broader clinical and imaging variables beyond the standard CRT response metrics. The MADIT-CRT Response Score was a landmark initiative that included parameters such as sex, prior heart failure hospitalization, LV end-diastolic volume, left atrial volume, cardiomyopathy etiology, and QRS duration and morphology (40). With a scoring range from 0 to 14, it demonstrated that patients in the highest quartile experienced up to a 69% relative reduction in the risk of death or HF compared to those in the lowest quartile. Similarly, the VALID-CRT score adopted a more comprehensive predictive index approach, integrating age, sex, diabetes status, NYHA class, LV EF, device type (CRT-P vs. CRT-D), whether the rhythm was atrial fibrillation and AV junction ablation was performed (41). While VALID-CRT was primarily designed to predict long-term outcomes post-CRT, both scores represent important steps toward multidimensional risk stratification beyond the current concepts and criteria. However, both scores were derived from retrospective registries and did not incorporate relevant biochemical markers, advanced imaging modalities, and pivotal LV parameters —such as myocardial strain and diastolic function indices—with proven prognostic value in recent studies (33, 34, 42). Furthermore, neither of these scores prioritized patient-perceived functional improvement as a primary objective, although arguably it is the most meaningful benefit from the patient perspective.

Overall, these efforts highlight the crucial need for integrating other relevant parameters into future predictive models to enhance CRT patient selection and better reflect patient-centered outcomes.

### Clinical implications and future directions

Findings of this study suggest that integrating biochemical markers (NT-proBNP, renal function), advanced imaging parameters (CMR feature tracking-derived strain), and diastolic function assessment could significantly improve CRT patient selection and lead to well-founded outcome expectations. These findings emerged despite standardized GDMT optimization across both patient groups, underscoring that the observed predictors represent intrinsic patient characteristics that may guide therapeutic decision-making independent from optimal GDMT. A multi-dimensional approach may better identify patients most likely to achieve functional benefits while avoiding unnecessary implantations in those unlikely to achieve meaningful gains.

Future research should focus on prospective validation of these predictive parameters in larger, diverse populations. Development of integrated risk scores or machine learning algorithms incorporating these multi-modal parameters could further enhance clinical utility. Investigation of whether tailored CRT programming based on these predictors improves outcomes represents another promising avenue.

### Study limitations

Several limitations in this study warrant consideration. This single-center retrospective design may limit generalizability, though our institution has fixed follow-up protocols for CRT recipients, which largely standardize assessments. The relatively small sample size (*n* = 97) and modest follow-up duration (median 36 months) may have limited detection of smaller effect sizes and decreased the sensitivity to capture late occurring responses. The absence of validated quality-of-life instruments, such as the Kansas City Cardiomyopathy Questionnaire (KCCQ) or Minnesota Living with Heart Failure Questionnaire, represents another limitation. While NYHA classification provides clinically meaningful assessment of functional capacity, complementary standardized QoL measures would have strengthened our patient-centered outcome evaluation. Although data on mortality and heart failure hospitalizations were collected during follow-up (showing no significant differences between groups), these were not primary endpoints, and our study was not powered for these analyses. Our focus remained on patient-perceived functional improvement as the most directly relevant outcome to individual patients. NYHA improvement was elected as the primary endpoint because it is one of the best representations of patient-centered benefits, however, its subjectivity might have allowed for reporting bias. The study population was predominantly male, requiring future studies to explore if there might be a gender-related difference in CRT response. Finally, this study indicates a promising benefit for the multi-parametric approach, yet prospective validation in larger, multi-center cohorts is essential before clinical implementation.

## Conclusion

Patient-centered functional improvement after CRT is best predicted by a combination of clinical, biochemical, and imaging parameters. Renal functions, baseline myocardial strain, and post-implantation LV diastolic function demonstrated robust predictive performance in HFrEF patients receiving CRT. Integration of these parameters may enhance CRT selection criteria and patient-centered response prediction beyond current conventional parameters. Prospective validation of the additive benefit of integrating these parameters into predictive models or machine learning algorithms are recommended.

## Data Availability

Data are available for sharing upon reasonable request to the corresponding author. Requests to access these datasets should be directed to ahmadsamir11@cu.edu.eg.
